# Finding of the factors affecting the severity of COVID-19 based on mathematical models

**DOI:** 10.1038/s41598-021-03632-x

**Published:** 2021-12-20

**Authors:** Jiahao Qu, Brian Sumali, Ho Lee, Hideki Terai, Makoto Ishii, Koichi Fukunaga, Yasue Mitsukura, Toshihiko Nishimura

**Affiliations:** 1 School of Integrated Design Engineering, Keio University, Yokohama, Kanagawa Japan; 2 Department of Neuropsychiatry, Keio University School of Medicine, 35 Shinanomachi, Shinjuku-ku, Tokyo, Japan; 3 Department of Anesthesia, Stanford University School of Medicine, Stanford, CA USA

**Keywords:** Computational science, Computer science, Scientific data, Predictive markers, Biomarkers, Computational models, Data mining, Machine learning

## Abstract

Since 2019, a large number of people worldwide have been infected with severe acute respiratory syndrome coronavirus 2. Among those infected, a limited number develop severe coronavirus disease 2019 (COVID-19), which generally has an acute onset. The treatment of patients with severe COVID-19 is challenging. To optimize disease prognosis and effectively utilize medical resources, proactive measures must be adopted for patients at risk of developing severe COVID-19. We analyzed the data of COVID-19 patients from seven medical institutions in Tokyo and used mathematical modeling of patient blood test results to quantify and compare the predictive ability of multiple prognostic indicators for the development of severe COVID-19. A machine learning logistic regression model was used to analyze the blood test results of 300 patients. Due to the limited data set, the size of the training group was constantly adjusted to ensure that the results of machine learning were effective (e.g., recognition rate of disease severity > 80%). Lymphocyte count, hemoglobin, and ferritin levels were the best prognostic indicators of severe COVID-19. The mathematical model developed in this study enables prediction and classification of COVID-19 severity.

## Introduction

In December 2019, a large number of viral pneumonia cases were noted in Wuhan, China. This pneumonia was found to be caused by a new type of coronavirus—severe acute respiratory syndrome coronavirus 2 (SARS-CoV-2)^1,2^, and the disease was named coronavirus disease 2019 (COVID-19)^3^. Subsequently, the disease was declared a pandemic and as of April 7, 2021, there were more than 132 million cases of COVID-19, with the death toll reaching 2.8 million worldwide^4^.

The symptoms of COVID-19 vary across patients^5^, and typical symptoms include fever, cough, and fatigue, while some individuals may also lose their sense of taste and smell. The severity of symptoms varies across individuals, and COVID-19 affects both the upper and lower respiratory tracts. The majority of individuals who are infected with SARS-CoV-2 develop mild-to-moderate cold-like symptoms or may even be asymptomatic. However, the probability of developing pneumonia is very high for elderly individuals.

Treatment of patients with severe COVID-19 is challenging. To date, to the best of our knowledge, no specific drug or treatment has been approved for COVID-19. Hydroxychloroquine was initially used for treatment during the early phase of the pandemic, but clinical trials subsequently showed that it was not effective^6^. Trials have also shown that dexamethasone is effective at reducing mortality in patients with severe COVID-19, but it had no effect on patients who did not require oxygen supplementation^7^. In a study by John et al.^8^, the recovery time of hospitalized patients with lower respiratory tract infection who received remdesivir was shorter than that of patients in the placebo control group. Among the 532 patients who received remdesivir treatment, 131 (24.6%) had serious adverse reactions. Remdesivir is currently the only drug that has been approved by the US Food and Drug Administration for treatment of COVID-19^9^. However, in a trial organized by the World Health Organization^10^, remdesivir as well as hydroxychloroquine, lopinavir, and interferon regimens did not affect the prognosis of hospitalized patients. Therefore, at present, there are no drugs or treatment methods that have proven to be completely effective for the treatment of COVID-19.

All patients with severe COVID-19 develop acute respiratory distress syndrome and frequently require mechanical ventilation to ensure sufficient oxygen supply, and some patients with severe COVID-19 may require extracorporeal membrane oxygenation (ECMO)^11,12^. Although ECMO can be life-saving in some cases, many complications are associated with the use of ECMO, including hemorrhage^13^, bacterial infections^14^ and stroke^15^. Thus, there is a need to reduce the number of severe COVID-19 cases that require such interventions.

To limit the development of severe COVID-19, effective preventative measures should be identified and implemented in care settings. For this, it is necessary to understand the factors that contribute to the development of severe COVID-19. A previous study attempted to develop logistic regression models for this purpose^16^; however, the results were not universally applicable due to the limited size of the data set used in the analysis.

Therefore, the present study aimed to expand upon previous work to develop more effective models for predicting COVID-19 disease severity by analyzing the blood test results of 300 COVID-19 patients according to disease severity using mathematical models and evaluating the prognostic factors in the model. Models designated seven factors that are known to affect the severity of COVID-19 as model variables and simultaneously compared the influence of each factor to determine its impact on patient outcome. This allowed for identification of the three most influential factors contributing to COVID-19 severity, which were then used for further analysis.

## Results

In the model generated when 30% of the available patient data (86 patients) were used for training the machine learning model, the ratio of the training group to the test group was 7:3. Mathematical formulae of the lasso, ridge, and logistic regression models are shown in Eqs. (1), (2), and (3), respectively. The results of the analysis using this training model are shown in Table 1.

**Table 1:** Clinical symptoms and signs according to disease severity (N = 312).

Symptom or sign	Disease severity	
	Non-severe (%) (N = 208)	Severe (%) (N = 104)
Altered level of consciousness	1	11
Fever (≥ 37.5 °C)	65	89
Cough	44	52
Sputum	16	24
Sore throat	19	13
Nasal discharge	14	1
Dysgeusia	22	13
Dysosmia	18	9
Shortness of breath	14	50
Diarrhea	10	16
Nausea and vomiting	2	9
Malaise	27	52
Bacterial infection	2	27
Fungal infection	0	5
Heart failure	0	13
Thromboembolism (including pulmonary embolism and cerebral infarction)	0	10
Liver dysfunction	4	39
Renal dysfunction	0	17
Macrophage activation syndrome (including hemophagocytic syndrome)	0	2

### Lasso regression model expression

1 $$ y = 0.15*{\text{Cre}} - 0.02*{\text{lymphocyte}}\,{\text{fraction}} + 0.08*{\text{LDH}} + 0.06*{\text{CRP}} + 0.10 $$

### Ridge regression model expression

2 $$ \begin{aligned} y & = 0.15*{\text{Cre}} - 0.03*{\text{Hb}} - 0.007*{\text{lymphocyte}}\,{\text{fraction}} \\ & \quad + 0.07*{\text{CRP}} + 0.08*{\text{LDH}} - 0.01*\left( { - 0.10} \right)*{\text{ferritin}} + 0.008*D - {\text{dimer}} + 0.1 \\ \end{aligned} $$

### Logistic regression model expression

3 $$ \begin{aligned} y & = 5.05*{\text{Cre}} - 0.28*{\text{Hb}} - 0.90*{\text{lymphocyte}}\,{\text{fraction}} \\ & \quad + 0.99*{\text{CRP}} + 2.21* {\text{LDH}} - 1.43*{\text{ferritin}} - 0.34*D - {\text{dimer}} - 4.62 \\ \end{aligned} $$

The regression equation using 100% of the learning data of all patients is shown in Eq. (4).

4 $$ \begin{aligned} y & = 0.29*{\text{Cre}} - 0.03*{\text{Hb}} - 0.07*{\text{lymphocyte }}\,{\text{fraction}} \\ & \quad + 0.08*{\text{CRP}} + 0.80* {\text{LDH}} - 0.68*{\text{ferritin}} - 0.30*D - {\text{dimer}} - 0.06 \\ \end{aligned} $$

where Cre, CRP, Hb, and LDH indicate creatinine, C-reactive protein, hemoglobin, lactate dehydrogenase, respectively.

## Discussion

The parameters in Eqs. (1), (2), and (3) are coefficients of each feature in each mathematical machine learning model. Using Eqs. (1), (2), and (3), we compared the coefficients of each impact factor, and the results of the comparisons are shown in Table 1.

Although the degree of influence of the prognostic factors varied in the different models, creatinine (Cre), lactate dehydrogenase (LDH), and C-reactive protein (CRP) were influential factors in all the three models. This result is consistent with results of previous research.^16^ We attempted to combine the results of previous studies to determine the reasons why these three prognostic indicators were so influential.

### Abnormal increase in Cre levels

Cre is a marker of kidney function. The results of the mathematical model showed that deterioration of renal function is related to COVID-19 severity. According to a study by Ishii et al.^17^, hyperuricemia and renal dysfunction are caused due to increased oxidative stress. Inflammation and oxidative stress are important indicators of COVID-19. Chronic kidney disease, which is associated with hyperuricemia, is likely to be a factor influencing COVID-19 severity, although the mechanism is unknown.

### Abnormal increase in LDH levels

In one study, many COVID-19 patients had abnormally elevated liver function marker levels^18^. LDH is a marker of liver function, and a study by Shailendra et al.^19^ found that the risk of death in patients with severe COVID-19 with liver dysfunction was threefold higher than that in patients without liver dysfunction and that the risk of death was increased 4.6-fold in individuals with cirrhosis. The angiotensin converting enzyme 2 receptor, by which SARS-CoV-2 gains entry to cells, is abundant in the liver and bile ducts where SARS-COV-2 infection can cause local damage. In the models to predict disease severity, we did not use other biochemical markers of liver function, such as aspartate aminotransferase, alanine aminotransferase, and gamma-glutamyl transpeptidase. A study by Kishaba et al.^20^ found that LDH was a marker of the severity of idiopathic pulmonary fibrosis. Further, Yan et al.^21^, found that an isolated increase in LDH levels without an increase in the other liver enzyme levels is indicative of inflammation (including COVID-19 pneumonia) and that this level is elevated as a result of tissue breakdown.

### Abnormal increase in CRP

Cytokine storms (a form of hyperimmune response) cause death in patients with severe COVID-19^22^. Elevated CRP level is a marker of an acute inflammatory response, and elevated lymphocyte levels are an indicator of infection with viruses and other pathogens and immune function^23^. The immune system over-responds in severe cases of COVID-19, causing systemic acute inflammation that is associated with more severe diseases.

As shown in Table 1, the predictive ability of the logistic regression model considerably exceeded that of the lasso and ridge models. Therefore, the logistic regression model should be used as the basis for comparing the degree of influence of each factor on the model. Data from only 300 patients was included in our study, and this ensured that the model recognition rate was high (recognition rate > 80%) by changing the size of the training group of the machine learning by the logistic regression model. Increasing the number of simulations allowed for comparison of different training groups in terms of the degree of influence of the following factors (the coefficient of each factor in the mathematical model). Our method for this comparison was as follows:

1. The size of the training group was set at 10% intervals to 10%, 20%, 30%, …… 90%, yielding a total of nine groups.
2. Twenty simulations were performed on the training data of each group.
3. After normalizing the coefficients (Z-score) of each factor obtained from each simulation, the value of the average of the 20 sets of results was calculated.

**Figure 1:**
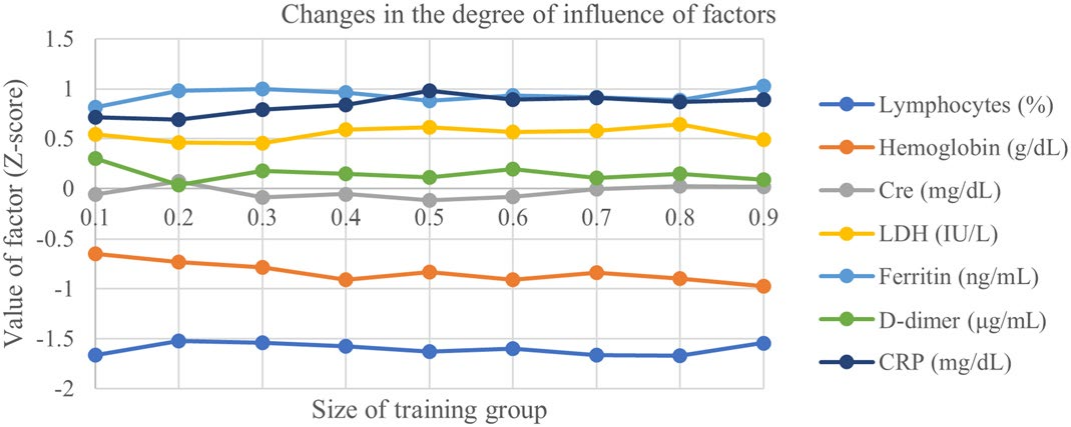
Association between the degree of influence of each factor according to the size of the training group. Cre, Creatine; CRP, C-reactive protein; LDH, lactate dehydrogenase.

**Figure 2:**
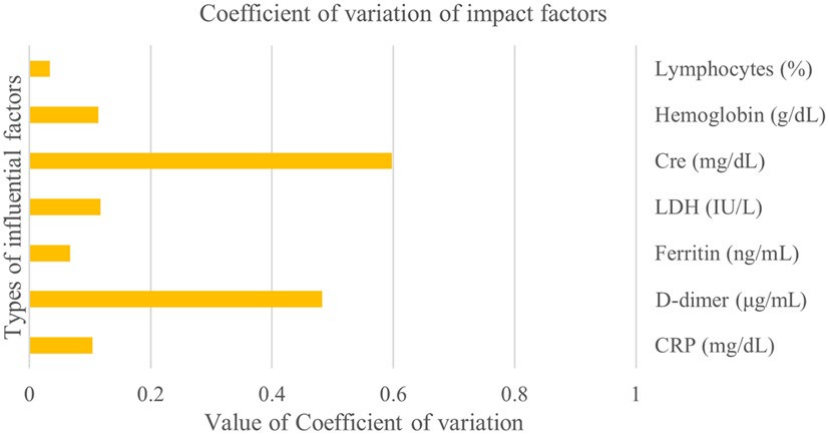
The coefficient of variation of each predictor in different training groups.

Figure 1 shows the results of these calculations. At the same time, we also calculated the coefficient of variation of the degree of influence of each factor, considering the absolute value, and the results are shown in Fig. 2.

As shown in Fig. 1, even if the amount of data in the training group changed significantly, there was very little change in the degree of influence of the factors. Based on the blood test results of 300 patients in our study, lymphocytes were the most influential factor, followed by ferritin, Hb, and CRP.

As shown in Fig. 2, the degree of change in lymphocytes was the smallest, followed by ferritin, whereas the degree of change in hemoglobin had a similar influence as that of LDH.

### Abnormal decrease in lymphocyte levels

Although the amount of patient data in this study was small, lymphocyte counts consistently had the greatest predictive value for disease severity. Lymphopenia is common in patients with COVID-19^24^. A study by Fajgenbaum and June^23^ showed that lymphopenia was not often present in diseases complicated by cytokine storms, but it is a marker of COVID-19 severity. However, it is not clear whether the lymphopenia observed in COVID-19 patients is due to tissue infiltration or lymphocyte destruction.

### Abnormal increase in ferritin levels

In another study^25^, an abnormal increase in ferritin levels was found in patients who required mechanical ventilation. This is consistent with the definition of disease severity in our study (using ventilator as one of the definitions), wherein an abnormal increase in ferritin level was identified as another marker of severe COVID-19.

### Abnormal decrease in hemoglobin levels

The oxygen saturation of hemoglobin determines the degree of hypoxia^24^. Most patients with severe disease needed supplemental oxygen to maintain an oxygen saturation above 90%. Therefore, a decrease in hemoglobin level is closely related to hypoxia. When there is a large reduction in Hb levels, patients will enter the hypoxemia state, which will affect the prognosis.

A machine learning logistic regression model was used to analyze the influence of blood biomarkers on COVID-19 severity using patient blood data. Cre, LDH, and CRP had the greatest influence on the model when the recognition rate of the model was maximum, whereas lymphocyte counts, ferritin, and hemoglobin consistently had a high influence on the model outcome when the number of simulations increased, while maintaining a high recognition rate.

In conclusion, the findings of these statistical models indicates that lymphocyte counts as well as ferritin and hemoglobin levels are important indicators of COVID-19 severity.

## Materials and methods

### Ethics and funding

This study was approved by the Ethics Committee of Keio University and performed in accordance with the Declaration of Helsinki. The study protocol was approved by the Ethics Review Board of Keio University (approval ID 20,200,055). The study was funded by Keio Research Global Institute (grant number: UMIN000041186).

### Patients

Patients with COVID-19 who were admitted to either Keio University Hospital or one of six community hospitals located in the greater Tokyo area (Saitama City Hospital, Tokyo Saiseikai Central Hospital, Federation of National Public Service Personnel Mutual Aid Associations, Tachikawa Hospital, National Hospital Organization Tokyo Medical Center, Nihon Koukan Hospital, and Saiseikai Utsunomiya Hospital) were included in the present study. In total, 312 patients meeting these criteria were identified, of whom, 12 patients were excluded due to incomplete data. Thus, patient data of 300 patients with COVID-19 were used in the present analysis.

Informed consent was obtained from all study subjects. Consent was obtained directly from adults aged ≥ 20 years. For minors aged < 20 years, consent was obtained both from the patient and from the patient’s parent or legal guardian.

Patients were divided into three major groups according to age: group 1 (0–30 years old), group 2 (31–60 years old), and group 3 (60 years old and above). Group 1 comprised 29 male patients (1 with severe COVID-19) and 44 female patients (1 with severe COVID-19), group 2 comprised 86 male patients (31 with severe COVID-19) and 45 female patients (7 with severe COVID-19), and group 3 comprised 63 male patients (40 with severe COVID-19) and 45 female patients (24 with severe COVID-19).

Patients who satisfied any of the following three criteria were defined as having severe COVID-19: 1) admitted to an intensive care unit, 2) required mechanical ventilation, or 3) died of COVID-19-related disease.

### Symptoms and signs

Direct symptoms of COVID-19 as well as types of complications associated with COVID-19 were summarized according to previous findings^26–30^. Accordingly, the following 19 items were assessed: 1. altered level of consciousness; 2. fever (≥ 37.5℃); 3. cough; 4. sputum production; 5. sore throat; 6. nasal discharge; 7. dysgeusia; 8. dyssomnia; 9. shortness of breath; 10. diarrhea; 11. nausea and vomiting; 12. malaise; 13. bacterial infection; 14. fungal infection; 15. heart failure; 16. thromboembolism (including pulmonary embolism and cerebral infarction); 17. liver dysfunction; 18. renal dysfunction; and 19. macrophage activation syndrome (including hemophagocytic syndrome). Patient records of the 300 study participants were reviewed for presence of the preceding 19 items, and association between these factors and COVID-19 severity is shown in Table 2.

**Table 2:** Accuracy of the three evaluation models and comparison of the influence of each factor in each regression model from greatest to least.

Regression type	Training group recognition rate	Test group recognition rate	1st	2nd	3rd	4th
Lasso	0.63	0.31	Cre	LDH	CRP	Hb
Ridge	0.64	0.3	Cre	Ferritin	CRP	LDH
Logistic	0.98	0.88	Cre	LDH	Ferritin	CRP

Cre, creatine; CRP, C-reactive protein; Hb, hemoglobin; LDH, lactate dehydrogenase.

From the results presented in Table 2, it is apparent that to reduce the incidence of each symptom in the non-severe group and the severe group separately, the difference may not exceed 50%. This is likely because the patient data were collected immediately after patient hospitalization; therefore, symptoms were likely to worsen as the disease developed further. In addition, patients with severe COVID-19 had a higher prevalence of most of the 19 factors analyzed in this study compared with patients with non-severe COVID-19. However, the number of patients with severe COVID-19 in this study was very small to verify whether these factors were generally predictive; therefore, early symptoms were not good predictors of disease severity in this study.

### Blood test results

We analyzed patient blood test results and selected predictors from the 35 biomarkers assessed in these tests. These selected factors were used to predict disease severity. First, we summarized the clinical signs of 30% of the patients (86 patients) who had similar clinical manifestations as the entire patient population. These clinical signs appear to be manifestations of concurrent changes in blood biochemical indexes, as reflected in blood test results. Additionally, we reviewed previous studies of patients with severe COVID-19 and summarized their results. Previous studies have shown that diabetes mellitus and hypertension, among other factors, are associated with greater disease severity^31^. In a study by Liu et al.^22^, the immune function of patients was closely related to disease severity. Further, Mohammad et al.^32^ reported that there was an interaction between liver function and the development of severe COVID-19.

Although previous studies have noted that age is an important indicator^33–35^, symptoms and age alone cannot help in appropriate classification of severe and non-severe COVID-19 patients during the early stages of COVID-19. Therefore, we decided to use patient blood test results to analyze the causes of severe COVID-19. In this analysis of 312 patients, the age of patients with severe disease patients was higher than that of patients with mild or moderate disease; however, we did not include age as a predictor of disease severity. As individuals get older, they are more likely to develop other underlying diseases, and their immune function declines. Thus, age has a strong influence on the development of severe disease, but its influence is indirect. Further, a study by Faust et al.^36^ showed that older individuals as well as young individuals aged 25–44 years have increased mortality rates. This supports our speculation that the influence of age on the development of severe COVID-19 is limited as well as our decision to solely analyze patient blood test results to identify predictors of disease severity.

A total of 35 blood biochemical indicators were assessed in study participants. Based on expert medical opinion by physician and combined with the results of previous COVID-19 studies, we designated seven blood indicators as variables in the mathematical model, namely (1) lymphocyte count (%)^37^; (2) hemoglobin (g/dL)^38^; (3) Cre (mg/dL)^39^; (4) LDH (IU/L)^40^; (5) ferritin (ng/mL)^41^; (6) D-dimer (μg/mL)^42^; and (7) CRP (mg/dL)^43^.

### Statistical methods

First, we assessed the results of three different mathematical models, namely a lasso regression model, ridge regression model, and logistic regression model. After comparing the results of these models, we decided to use the logistic regression model for the classification of COVID-19 severity using 30% of the available patient data for machine learning purposes. Additionally, we used lasso^44^ and ridge regression^45^ models to compare with the machine learning results obtained using the logistic regression model.

Finally, the resulting logistic regression model of classification was used to compare the influence of each factor on COVID-19 severity. The sample size of this study is small; therefore, when comparing the influence degree of each factor, it is possible that the results obtained will vary greatly. To reduce the impact of sample size on the results, we decided to change the size of the logistic regression model training group and increase the number of simulations to ensure accuracy of the model. To ensure that the classification accuracy of disease severity was > 80%, the data size of the training group for each iteration of this process was changed (e.g. training group: 10%, 20%…, 90% of the total data volume), and the results of each simulation were then averaged.

## Data Availability

Data and code are available from the corresponding author.
